# Use of age-dependent FRAX-based intervention thresholds for Singapore

**DOI:** 10.1007/s11657-020-00782-9

**Published:** 2020-07-22

**Authors:** John A. Kanis, Manju Chandran, Siok Bee Chionh, Ganga Ganeson, Nicholas C Harvey, Woon-Puay Koh, Timothy Kwok, Tang Ching Lau, Enwu Liu, Mattias Lorentzon, Eugene V McCloskey, Kelvin Bryan Tan, Liesbeth Vandenput, Helena Johansson

**Affiliations:** 1 Centre for Metabolic Bone Diseases, University of Sheffield, Beech Hill Road, Sheffield, S10 2RX UK; 2 Mary McKillop Institute for Health Research, Australian Catholic University, Melbourne, Australia; 3 Osteoporosis and Bone Metabolism Unit, Department of Endocrinology, Singapore General Hospital, Singapore, Singapore, Singapore; 4 Department of Medicine, Yong Loo Lin School of Medicine, National University of Singapore, Singapore, Singapore; 5 Division of Policy, Research and Evaluation, Ministry of Health, Singapore, Singapore; 6 MRC Lifecourse Epidemiology Unit, University of Southampton, Southampton, UK; 7 Health Services and Systems Research, Duke-NUS Medical School, 8 College Road, Singapore, 169857 Singapore; 8 Saw Swee Hock School of Public Health, National University of Singapore, 12 Science Drive 2, Singapore, 117549 Singapore; 9 Department of Medicine and Therapeutics, Prince of Wales Hospital, The Chinese University of Hong Kong, Shatin, Hong Kong, China; 10 Jockey Club Centre for Osteoporosis Care and Control, The Chinese University of Hong Kong, Shatin, Hong Kong, China; 11 Department of Medicine, Yong Loo Lin School of Medicine, National University of Singapore, Singapore, Singapore; 12 Geriatric Medicine, Department of Internal Medicine and Clinical Nutrition, Institute of Medicine and Clinical Nutrition, Sahlgrenska Academy, University of Gothenburg, Gothenburg, Sweden; 13 MRC and Arthritis Research UK Centre for Integrated Research in Musculoskeletal Ageing, Mellanby Centre for Bone Research, University of Sheffield, Sheffield, UK; 14 Ministry of Health Singapore, Singapore, Singapore; 15 Department of Internal Medicine and Clinical Nutrition, Institute of Medicine, Sahlgrenska Academy, University of Gothenburg, Gothenburg, Sweden

**Keywords:** Osteoporosis, Fracture risk assessment, FRAX, Intervention threshold

## Abstract

***Summary*:**

Assessment and treatment pathways based on age-specific intervention thresholds in Singapore using FRAX paths can be used to identify patients at high risk of fracture and avoid unnecessary treatment in those at low risk.

**Purpose:**

Intervention thresholds for the treatment of osteoporosis have been based historically on the measurement of bone mineral density. The development of FRAX® has permitted a more accurate assessment of fracture risk. The aim of the present study was to explore treatment paths and characteristics of women selected for treatment in Singapore based on FRAX.

**Methods:**

The approach to the setting of intervention and assessment thresholds used the methodology adopted by the National Osteoporosis Guideline Group for FRAX-based guidelines in the UK but based on the epidemiology of fracture and death in Singapore. The methodology was applied to women age 50 years or more drawn from the population-based Singapore Chinese Health Study (SCHS) cohort. Missing data for the calculation of FRAX was simulated using data from Chinese cohorts from Hong Kong.

**Results:**

Intervention thresholds expressed as a 10-year probability of a major osteoporotic fracture ranged from 2.9% at the age of 50 years increasing to 32% at the age of 90 years. A total of 1927 of 29,323 women (7%) had a prior fragility fracture and would be eligible for treatment for this reason. An additional 3019 women (10.3%) would be eligible for treatment on the basis of age-dependent thresholds. The mean BMD T-score of women so selected was −2.94.

**Conclusion:**

Probability-based assessment of fracture risk using age-specific intervention thresholds was developed for Singapore to help guide decisions about treatment.

## Introduction

Osteoporosis is a common, chronic, and costly condition; the annual economic burden in Singapore associated with fragility fractures was estimated at approximately € 118 million in 2017 and is forecast to increase to € 186.9 million by 2035 [1]. In Europe, the annual cost of fractures associated with osteoporosis exceeded € 37 billion in 2010 [2]. Disability due to fragility fractures was greater than that caused by any single cancer, with the exception of lung cancer and was comparable or greater than that caused by a variety of chronic noncommunicable diseases, such as rheumatoid arthritis-, asthma-, or high blood pressure-related heart disease [3]. Fortunately, a wide range of treatments is available that improve bone mass and decrease the risk of fractures associated with osteoporosis [4]. The use of such interventions by healthcare practitioners is assisted by instruments that assess patients’ fracture risk to optimize clinical decisions about prevention and treatment. The most widely used web-based tool FRAX® (https://www.sheffield.ac.uk/FRAX/) meets these requirements and computes the 10-year probability of fragility fractures based on several common clinical risk factors and, optionally, a bone densitometry result obtained from dual x-ray absorptiometry (DXA) [5, 6]. FRAX models are available for 66 countries covering more than 80% of the world population at risk [7] and have been incorporated into more than 100 guidelines worldwide [8].

A country-specific FRAX model was developed for Singapore which was launched in December 2010. Whereas the model should enhance accuracy of determining fracture probability among the Singaporean population, guidance is not yet available to make decisions about treatment [9]. The aim of the present study was to explore a potential assessment pathway for treatment and characteristics of women selected for treatment in Singapore based on FRAX.

## Methods

### Population sample

The population sample used to determine the impact of intervention and assessment thresholds was drawn from the Singapore Chinese Health Study (SCHS). Details of the study cohort have been previously described [10, 11]. In brief, the cohort was recruited between 1993 and 1998, drawn from permanent residents or citizens of Singapore who lived in government-built housing (86% of the Singapore population resided in such facilities at the time of recruitment, a proportion that has remained stable over time). Men and women of Chinese ethnicity age 45–74 years were eligible for inclusion. A total of 63,257 persons (∼85% of eligible and invited subjects) was enrolled. The present analysis was restricted to women age 50 years or more at recruitment (*n* = 29,323). Women were followed for an average of 9.1 years with a maximum of 11.5 years, and incident hip fractures were recorded.

Age and data on body mass index was available in all women. With regard to the dichotomous FRAX variables, information was available for prior fragility fracture (hip or other bone fractures), current smoking, secondary osteoporosis (prevalent type II diabetes only), and high alcohol intake (3 or more units per day). BMD values were not available nor were a parental history of hip fracture, exposure to glucocorticoids, and information on rheumatoid arthritis. For the purposes of this analysis, these variables were simulated.

### Simulation of Variables

Data from the Mr. and Ms. Os Hong Kong cohorts were used to identify appropriate logistic regression equations needed to generate data for the missing risk factors in the SCHS cohort using methods described previously [12–14]. Mr. and Ms. OS Hong Kong included Chinese men and women age 65 years and older who were recruited between 2002 and 2003. The cohort was age-stratified having 33% of subjects in each of the following age groups: 65–69, 70–74, and > 75 years. Subjects were recruited from housing estates and community centres for the elderly. Participants had BMD measured using Hologic QDR 4500 devices [15].

Logistic regression (for dichotomous risk factors) was used to examine the conditional probability of the association of the risk factor to be simulated for SCHS with age, sex, and body mass index (BMI) as continuous variables and with previous fracture, current smoking, and alcohol intake as dichotomous variables. For family history of hip fracture, the associations between the variable and age and previous fracture were used. For glucocorticoid use, the associations between the variable and age, sex and BMI were used. For rheumatoid arthritis, the associations between the variable and sex, BMI and previous fracture were used. Since the weight of the dichotomous clinical risk factors is similar in men and women, the logistic regressions were determined from Mr. and Ms. OS combined to provide greater power to determine the logistic regressions to be used.

The equations identified in the logistic regressions for the dichotomous risk factors were then applied to the measured risk factor data in the SCHS cohort to predict the likelihood of having a positive value for the missing key risk factor for each individual. Next, a random number was generated using a computer programme, which was then compared with the predicted likelihood for that variable for that individual. If the random number was less than or equal to the predicted probability, the woman was assigned a positive value for the risk factor. If the random number was greater than the predicted probability, the woman was assigned a negative value for the risk factor. In this way FRAX-based fracture probabilities (without BMD) could be computed for the SCHS cohort. The adequacy of the simulations was checked by comparing the observed number of hip fractures with those predicted from hip fracture probabilities computed by FRAX. In addition, the prevalence of the simulated variables was compared with the age-adjusted prevalence from Ms. Os.

The simulations for femoral neck BMD were based on examining the conditional probability of the association of BMD with risk factors, age, and BMI, by linear regression [13]. For BMD, the associations between the variable and age, BMI, previous fracture, and smoking were used. We tested the validity of the simulation by computing the sensitivity and specificity of the Osteoporosis Self-Assessment Tool for Asians (OSTA) that is used in Singapore to identify women with osteoporosis from height and weight [16]. We additionally compared the age-matched BMD values in the Ms. Os and simulated cohort.

### Fracture Probabilities

The 10-year probabilities of hip fracture and a major osteoporotic fracture (clinical spine, hip, humerus, or distal forearm fracture) were calculated using the FRAX model for Singapore (web version 4.1). Calculations were undertaken with and without the inclusion of femoral neck BMD.

### Intervention Thresholds Based on FRAX

The use of FRAX in clinical practice demands a consideration of the fracture probability at which to intervene, both for treatment (an intervention threshold) and for BMD testing (assessment thresholds). The approach to the setting of intervention and assessment thresholds used the methodology adopted by the National Osteoporosis Guideline Group for FRAX-based guidelines in the UK [17, 18].

A criterion for recommending intervention in women is a history of a prior fragility fracture since many guidelines recommend that postmenopausal women with such an event may be considered for intervention without the necessity for a BMD test (other than to monitor treatment) [4, 8, 9, 18–22]. Given that a prior fragility fracture is considered to carry a sufficient risk to recommend treatment, the intervention threshold in women without a prior fragility fracture can be set at the age-specific 10-year probability of a major osteoporotic fracture (hip, spine, forearm, or humerus) equivalent to women with a prior fragility fracture using the FRAX model for Singapore. Body mass index was set at an ethnic- and age-dependent value [23, 24].

The age-specific 10-year probability of a major osteoporotic fracture equivalent to women with a prior fragility fracture was calculated for each ethnicity in Singapore. Then an intervention threshold was calculated using these probabilities weighted by the ethnic-specific population of Singapore from 2017 at each 5-year interval from the age of 40 years [25]. The setting of the intervention threshold differed from a previous estimate by using age-specific data for the ethnic composition of the population rather than a single estimate for all ages [24].

### Assessment thresholds for BMD testing.

Two assessment thresholds for making recommendations for the measurement of BMD were considered [17, 18]:

- A threshold probability below which neither treatment nor a BMD test should be considered (lower assessment threshold).
- A threshold probability above which treatment may be recommended irrespective of BMD (upper assessment threshold).

The lower assessment threshold was set to exclude a requirement for BMD testing in women without clinical risk factors, as given in current European guidelines [4, 19, 20]. It was therefore set to the age-specific 10-year probability of a major fracture equivalent to women with no clinical risk factors. An upper threshold was chosen to minimize the probability that a patient, characterized to be at high risk using clinical risk factors alone, would be reclassified to be at low risk with additional information on BMD and vice versa [26]. The upper assessment threshold was set at 1.2 times the intervention threshold as used in the UK [17].

### Assessment strategy

As noted above, women with a prior fragility fracture were considered to be eligible for treatment without the need for further assessment. In women without a previous fragility fracture, the management strategy was based on the assessment of the 10-year probability of a major osteoporotic fracture (clinical spine, hip, forearm, or humerus). Women with probabilities below the lower assessment threshold were not considered eligible for treatment. Women with probabilities above the upper assessment threshold were eligible for treatment. Women with probabilities between the upper and lower assessment thresholds were to be referred for BMD measurements and their fracture probability reassessed. On reassessment of FRAX with the inclusion of femoral neck BMD, individuals were considered eligible for treatment when fracture probabilities lay above the intervention threshold.

## Results

The baseline characteristics are given in Table 1. The prevalence of the simulated variables was similar to the age-matched prevalence of these risk factors from Ms. Os (Table 4, Appendix A). Similarly, age-matched BMD values in the SCHS cohort were similar to those in the Ms. Os cohort (Table 5, Appendix A). In the SCHS cohort, the Osteoporosis Self-Assessment Tool for Asians (OSTA) yielded a sensitivity of 85% and specificity of 50% based on BMD at the femoral neck.

**Table 1:** Summary description of the baseline variables in SCHS cohort for women age 50 years or more (*N* = 29,323)

	N	Mean	SD	*n* (%)
Age (years)	29,323	61.7	7.8	
BMI (kg/m^2^)	29,323	23.2	3.6	
Femoral neck BMD (T-score)^2^	29,323	−1.89	0.87	
Previous fracture	29,323			1927 (6.6%)
Current smoking	29,323			1584 (5.4%)
Secondary osteoporosis ^1^	29,323			4228 (14.4%)
Alcohol 3 or more units per day	29,323			17 (0.0%)
Parental history of hip fracture^2^	29,323			1044 (6.6%)
Glucocorticoid exposure^2^	29,323			116 (0.4%)
Rheumatoid arthritis^2^	29,323			558 (1.9%)
Ten-year probability				Range
Hip fracture probability calculated without BMD		2.8	4.0	0.1–70.1
Hip fracture probability calculated with BMD		3.0	4.7	0.1–76.0
MOF probability calculated without BMD		7.5	6.7	0.9–77.7
MOF probability calculated with BMD		8.0	7.3	0.9–77.8

^1^Type 2 diabetes

^2^Simulated variable. *MOF* major osteoporotic fracture

Individual probabilities of hip fracture and a major osteoporotic fracture (with and without BMD) are given in Table 1. The mean probability of a major fracture was 7.5% and, for a hip fracture, was 2.8% when calculated without BMD. Probabilities calculated with BMD were similar. As expected, average fracture probabilities increased progressively with age. For a major osteoporotic fracture, the 10-year probability rose from 2.1% in the age category 50–54 years to 24.3% for the ages 80–84 years.

**Fig. 1:**
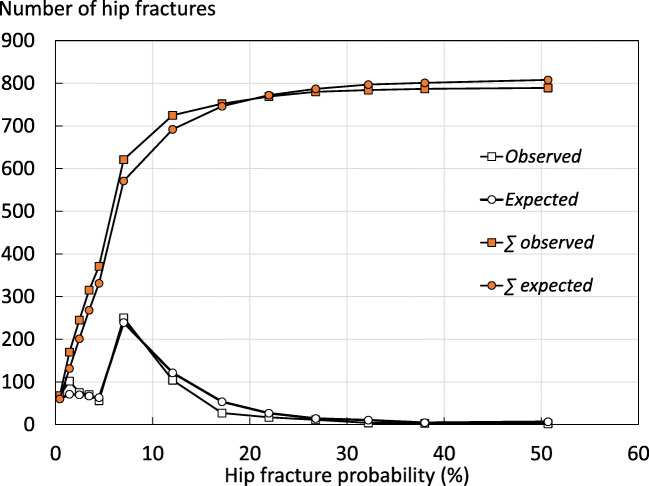
Number and cumulative number of hip fractures expected from categories of FRAX measured at baseline and hip fractures observed during follow-up. Intervals are closed to the left, (i.e. 0–1 = > 0–0.99, 1–2 = > 1–1.99)

Hip fracture incidence was recorded during an average of 9.1 years with a maximum of 11.5 years of follow-up. During this period with 266,025 person years of observation, 789 women experienced a first hip fracture (2.7%). FRAX-based hip fracture probabilities predicted 808 hip fractures. There was a close correspondence between FRAX-based hip fracture probability and observed hip fracture rates (Fig. 1).

### Thresholds

**Fig. 2:**
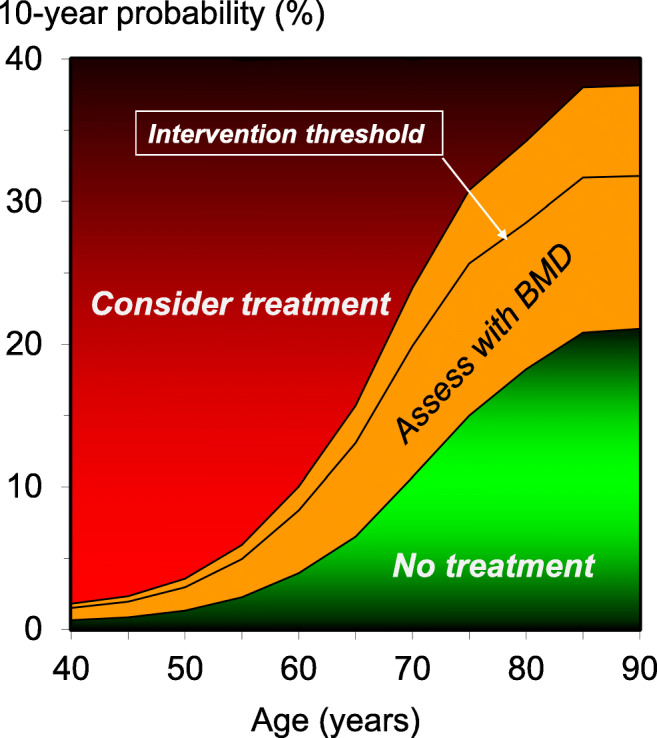
10-year probability (%) of a major osteoporotic fracture corresponding to the lower assessment threshold (LAT) and upper assessment threshold (UAT) for Singapore. The red area is where the treatment would normally be recommended, the orange area shows the limits of fracture probabilities for the assessment of BMD, and the green area is where treatment would not normally be recommended

The intervention threshold (set at the age-specific major osteoporotic fracture probability equivalent to women of average BMI with a prior fragility fracture) rose with age from a 10-year probability of 2.96% at the age of 50 years to 32% at the age of 90 years (Table 2 and Fig. 2). Table 2 and Fig. 2 also give the age-specific upper and lower assessment thresholds for recommending the measurement of BMD in the assessment of fracture probability. At the age of 65 years, for example, a BMD test would not be recommended in an individual with a fracture probability below 6.5%. At the same age, a BMD test would be recommended with a fracture probability that lay between 6.5 and 16%. Treatment would be recommended without the requirement of a BMD test (for fracture risk assessment, though possibly for monitoring of treatment) in individuals with a fracture probability that exceeded 16%. In individuals in whom a BMD test was undertaken and BMD entered to the FRAX calculation, treatment would be recommended in those with a fracture probability that was 13% or greater.

**Table 2:** Ten-year probability of a major osteoporotic fracture (%) by age at the intervention threshold and lower and upper assessment thresholds calculated with FRAX for Singapore adjusted for ethnicity

Age (years)	Intervention threshold^a^ (%)	Lower assessment threshold^b^ (%)	Upper assessment threshold^c^ (%)
40	1.51	0.65	1.81
45	1.95	0.85	2.34
50	2.96	1.32	3.56
55	4.94	2.26	5.93
60	8.35	3.95	10.02
65	13.07	6.51	15.68
70	19.87	10.68	23.85
75	25.67	14.99	30.80
80	28.53	18.24	34.23
85	31.66	20.81	37.99
90	31.79	21.08	38.15

^a^The threshold is the probability of a major osteoporotic fracture for a woman with a previous fracture and no other clinical risk factors without BMD

^b^The lower assessment is the probability of a major osteoporotic fracture for a woman with no clinical risk factors without BMD

^c^The upper assessment was set at 1.2 times the intervention threshold

### Management pathway

**Fig. 3:**
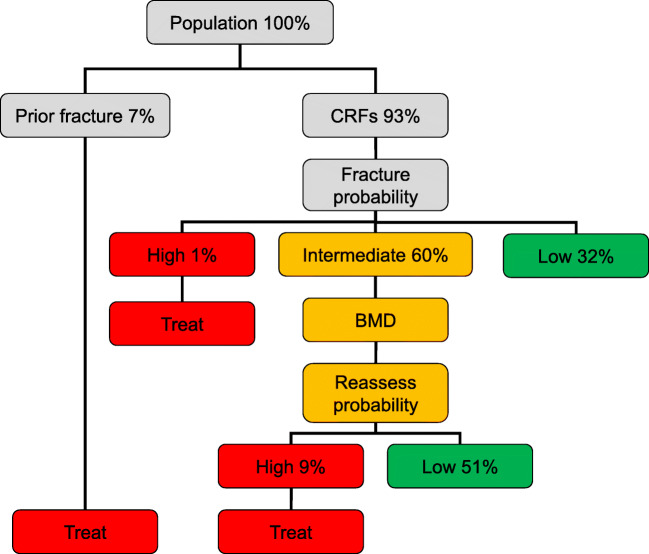
Management algorithm for the assessment of individuals at risk of fracture. The numbers in each category of risk denote the percentage of women in each category

One thousand nine hundred and twenty-seven of 29,323 women (7%) had a prior fragility fracture and would be eligible for treatment on this basis. For those without a prior fragility fracture (*n* = 27,396), the outcome of risk assessment is shown in Fig. 3. Of the whole cohort, 269 additional women (1%) would be eligible for treatment in that their fracture probability exceeded the upper assessment threshold for Singapore. Conversely, 9406 low risk women (32%) would not normally be eligible for further assessment in that their fracture probability lay below the lower assessment threshold. The intermediate category of risk in Fig. 2 comprised of 17,721 women (60%) in whom FRAX would be recalculated with the inclusion of femoral neck BMD. With the inclusion of BMD, 14,971 women were categorized at low risk (51% of the total cohort) and 2750 (9%) categorized at high risk. The overall disposition of the cohort is shown in Table 3. Those identified as eligible for treatment because of a prior fragility fracture or for a high FRAX score had higher fracture probabilities than those not eligible for treatment. The average 10-year fracture probability (calculated without BMD) in all women identified as eligible for treatment was 11.8 and 5.1% for major osteoporotic fracture and hip fracture, respectively.

**Table 3:** Disposition of the Singaporean cohort according to NOGG guidance

Category	Number	%	Mean BMD T-score	Mean 10-year probability without BMD (FRAX) (%)		Mean 10-year probability with BMD (FRAX) (%)	
				MOF	Hip	MOF	Hip
Entire cohort	29,323	100	−1.89	7.5	2.8	8.0	3.0
Treated (prior fragility fracture)	1927	6.6	−2.18	15.2	6.5	15.1	6.3
Otherwise eligible for treatment*	3019	10.3	−2.94	9.8	4.3	15.3	8.5
BMD tests	17,721	60.4	−1.98	7.7	2.9	8.2	3.2
No treatment	24,377	83.1	−1.74	6.6	2.3	6.5	2.0

*No prior fragility fracture but high FRAX score

## Discussion

In this report, we present intervention thresholds and BMD assessment thresholds based on fracture probability using FRAX. The approach used was similar to that adopted by the National Osteoporosis Guideline Group (NOGG) in the UK and more recently in European guidelines [4, 6, 18] but applied to the FRAX model for Singapore. Thus, the intervention threshold was set at the fracture probability equivalent to a woman from Singapore with a prior fragility fracture. The rationale is that if women with a prior fragility fracture are considered eligible for treatment, as commonly considered and, indeed, recommended in the present Singapore guidance [9], then women without fracture but with equivalent probabilities should also be eligible for treatment. The use of a single intervention threshold, despite ethnic-specific FRAX models, is appropriate in the sense that intervention is recommended at an identical fracture probability irrespective of ethnicity.

The starting point in the assessment of women is the presence of a clinical risk factor that alerts the physician to consider osteoporosis. The opportunistic case finding strategy arises because screening the general population is not widely recommended in Asia or Europe, though advocated in North America [12, 21]. In those eligible for assessment and in common with the NOGG guidelines, we limited the use of BMD testing. Individuals with fracture probabilities equivalent to, or lower than, those of women with no clinical risk factors (as used in FRAX) would not be assessed by BMD. At the other extreme, BMD testing was not universally recommended in individuals at high risk. The rationale is that reclassification of risk with the addition of a BMD test (from high risk to low risk and vice versa) is high when fracture probabilities estimated without BMD are close to the intervention threshold, but the likelihood of reclassification decreases the further away the probability estimate is from the intervention threshold [26]. The approach used has been well validated in the UK and Canada [14, 26–29].

The attraction of this approach is that this makes efficient use of BMD resources. The strategy implies, however, that patients at high risk, but identified without BMD, would respond to pharmacological intervention. The evidence that such patients respond to treatment is strong [29–35]. A principal reason is that BMD values are low in patients identified with FRAX but without a BMD test [29]. Overall, the mean T-score in women eligible for treatment and selected with FRAX was −2.94 (see Table 3).

In the present study, we have focused on intervention thresholds based on 10-year probabilities of a major osteoporotic fracture. There is, in principle, no reason why a strategy should not be based on the probability of hip fracture. Indeed, screening on this basis has recently been shown to decrease the incidence of hip fracture in the UK [36]. We have also assumed that measurements of BMD are included in the strategy. Where facilities for BMD testing are wanting, FRAX without BMD provides similar predictive value as BMD without FRAX [37]. Nevertheless, the combination of FRAX with BMD where appropriate provides the optimal strategy.

The implementation of this strategy is expected to lessen unnecessary treatment of individuals at low fracture risk and better direct treatments to those at high risk than treatment decisions based only on the measurement of BMD [37]. Implementation will, however, raise immediate problems in that current guidance for treatment in Singapore, and many other countries, is led by measurement of BMD. For example, patients are eligible for treatment with a T-score of −2.5 SD or lower. Thus, it will be important that healthcare agencies are involved in any implementation process.

There are a number of potential limitations of the present study to consider. First, although the cohort was large, it may not be representative of the Singaporean population. A recruitment bias towards healthier individuals is expected to preferentially lower fracture probabilities when BMD is included in the FRAX calculation. It is of interest that fracture probabilities were very similar when calculated with or without BMD, supporting a view that such bias is likely to be small. A more robust argument that biases were small was the close agreement between hip fracture incidence in the SCHS cohort and that predicted from the Singaporean FRAX model. Unfortunately, other outcome fractures were not available from the SCHS cohort to check the predictive value of probability estimates of a major osteoporotic fracture. Another important limitation was that not all FRAX variables were documented in the SCHS cohort and the missing values were simulated using regression equations derived from a Chinese cohort in Hong Kong. The adequacy of the simulations is supported by the similar prevalence of clinical risk factors in the Hong Kong and SCHS cohorts. Moreover, we tested the validity of the simulation of BMD by computing the sensitivity and specificity of OSTA. The sensitivity of 85% and specificity of 50% were very comparable with published estimates of 91 and 45%, respectively [16]. Meta-analyses of studies evaluating OSTA in Caucasian populations using the same cut off threshold of < 1 to identify postmenopausal women with osteoporosis at the femoral neck provided summary sensitivity and specificity estimates of 89% (95%CI 82–96%) and 41 (95%CI 23–59%), respectively [38]. These considerations suggest that the SCHS cohort was representative of the Singaporean population and that the treatment pathways are applicable to the general population.

The present study has shown that it is possible to apply FRAX-based assessment guidelines using the same principles that have been applied to guidelines elsewhere but tailored to the epidemiology of Singapore. The approach to intervention thresholds is based on the principles of case finding and does not consider a health economic perspective. Although the approach has been shown to be cost-effective in a UK setting [39], cost-effectiveness will necessarily differ in the context of Singapore because of different fracture risks and costs. It will be important therefore to underpin these guidelines with an economic assessment. Overcoming these hurdles will, however, improve the delivery of healthcare to those most at need.

## Data Availability

All data used to support the results of this study are stored at and available from the corresponding author upon request. Written, informed consent was not required for this study.

## Appendix

**Table 4:** The prevalence of the simulated risk factors for SCHS compared with the age-matched prevalence from Ms. Os

	Women in SCHS		Women in Ms. Os	
	N	Percentage (%)	N	Percentage (%)
**Age 65–69**				
Family history of hip fracture	4687	6	565	8
Corticosteroid use	4687	0	669	0
Rheumatoid arthritis	4687	2	669	2
**Age 70–74**				
Family history of hip fracture	3371	5	542	6
Corticosteroid use	3371	0	664	0
Rheumatoid arthritis	3371	2	665	2
**Age 75–79**				
Family history of hip fracture	1905	3	354	2
Corticosteroid use	1905	0	449	0
Rheumatoid arthritis	1905	2	449	2
**Age 80–84**				
Family history of hip fracture	257	2	125	2
Corticosteroid use	257	0	159	1
Rheumatoid arthritis	257	2	159	1

**Table 5:** Mean femoral neck BMD T-score and standard deviation (SD) by age for SCHS compared with Ms. Os

	Women in SCHS			Women in Ms. Os		
	n	Mean	SD	n	Mean	SD
Age 65–69	4687	−2.1	0.8	669	−2.0	0.8
Age 70–74	3371	−2.3	0.8	665	−2.3	0.8
Age 75–79	1905	−2.6	0.8	449	−2.5	0.8
Age 80–84	257	−2.7	0.9	159	−2.8	0.8
